# Varying Viral Replication and Disease Profiles of H2N2 Influenza in Ferrets Is Associated with Virus Isolate and Inoculation Route

**DOI:** 10.1128/jvi.00732-22

**Published:** 2022-07-11

**Authors:** Koen van de Ven, Harry van Dijken, Wenjuan Du, Femke de Heij, Justin Mouthaan, Sanne Spijkers, Sharon van den Brink, Paul Roholl, Cornelis A. M. de Haan, Jørgen de Jonge

**Affiliations:** a Centre for Infectious Disease Control, National Institute for Public Health and the Environment (RIVM), Bilthoven, the Netherlands; b Section Virology, Division Infectious Diseases & Immunology, Department Biomolecular Health Sciences, Faculty Veterinary Medicine, Utrecht University, the Netherlands; c Microscope Consultancy, Weesp, the Netherlands; Emory University School of Medicine

**Keywords:** animal models, infectious disease, influenza

## Abstract

H2N2 influenza virus, the causative agent of the 1957 “Asian flu” pandemic, has disappeared from circulation. However, H2-influenza viruses are still circulating in avian reservoirs. Combined with the waning of H2N2-specific immunity in the human population, there is a risk of reintroduction of H2N2 influenza virus. Vaccines could help in preventing a future pandemic, but to assess their efficacy animal models are required. We therefore set out to expand the ferret model for H2N2 influenza disease by infecting ferrets intranasally or intratracheally with four different H2N2 viruses to investigate their influence on the severity of disease. The H2N2 viruses were collected either during the pandemic or near the end of H2N2 circulation and covered both clade I and clade II viruses. Infection of ferrets with the different viruses showed that viral replication, disease, and pathology differed markedly between virus isolates and infection routes. Intranasal inoculation induced a severe to mild rhinitis, depending on the virus isolate, and did not lead to lung infection or pathology. When administered intratracheally, isolates that successfully replicated in the lower respiratory tract (LRT) induced a nonlethal disease that resembles that of a moderate pneumonia in humans. Differences in viral replication and disease between viruses could be associated with their binding preference for α2,3- and α2,6-sialic acid. The model presented here could facilitate the development of a new generation of H2N2 influenza vaccines.

**IMPORTANCE** In 1957 the world was subjected to a pandemic caused by an influenza A virus of the subtype H2N2. Although the virus disappeared in 1968, H2 viruses continue to circulate in avian reservoirs. It is therefore possible that the H2N2 influenza virus will be reintroduced into the human population, which can lead to another pandemic. The impact of a new H2N2 influenza pandemic can be mitigated by vaccination. However, these vaccines first need to be developed and tested in animal models. In preparation for this, we expanded the ferret model to mimic the different facets of human H2N2 influenza infection and disease. This model can be used for the development and evaluation of new H2N2 influenza vaccines.

## INTRODUCTION

In 1957 the “Asian flu” became the second influenza pandemic of the 20th century. The cause of the pandemic was an H2N2 influenza A virus, which resulted from a reassortment between avian influenza H2, N2, and PB1 genes with human H1N1 influenza gene segments (1–4). The virus quickly spread throughout the immune-naive population, leading to an estimated 1–2 million deaths during the pandemic (5, 6). After its introduction, H2N2 remained circulating as a seasonal influenza virus until it was replaced by H3N2 in 1968. Despite its disappearance, we are not safeguarded against a new introduction as genetically similar strains are still circulating in birds (7, 8) and people born after 1968 do not possess H2-neutralizing antibodies (6). With the pandemic track record of H2N2, this poses a risk now that humoral immunity against H2N2 on the population level is rapidly declining (6, 9).

Mutations in the receptor binding domain of hemagglutinin (HA) can affect its binding affinity to the receptor on cells. Avian-originating HA proteins are more likely to bind to α2,3-linked sialic acid (SA), while HA from human-adapted influenza strains prefer α2,6-SA (reviewed in reference 10). In the adaptation from avian to human hosts, binding-preference switching from α2,3-SA to α2,6-SA is likely an essential process for avian derived influenza viruses. Not surprisingly, most pandemic influenza A viruses with an HA of avian origin started circulating among humans with a mixed α2,3-SA and α2,6-SA binding preference (reviewed in reference 11). Continued circulation of these strains in the human population lead to a gradual increase in their binding preference for α2,6-SA. Importantly, α2,3-SA is mainly present on alveolar cells in the lower respiratory tract (LRT) of humans, while cells expressing α2,6-SA are primarily present in the upper respiratory tract (URT; reviewed in reference 10). URT infections are usually limited to symptoms of a common cold, while LRT infections can lead to severe pneumonia. A switch in binding affinity from α2,3- to α2,6-SA is thus often accompanied by lower disease burden. Hence, the binding preference of individual H2N2 strains might also influence their pathogenesis.

With the threat of a next H2N2 pandemic, animal models are required to evaluate H2N2 vaccines. In general, the ferret is considered the best small animal model to study protection against influenza due to its similarities to human influenza disease (12). This resemblance might be partly explained by the distribution of α2,6-SA, which is similar between ferrets and humans (13). Others have already shown that ferrets are effectively infected upon intranasal (i.n.) inoculation with H2N2 influenza (14–17), but not how disease and pathology are affected by intratracheal (i.t.) inoculation. By depositing influenza virus into the lungs via i.t. inoculation, more severe influenza disease can be modeled (18–20). We infected ferrets i.n. or i.t. with various pandemic and seasonal human H2N2 virus isolates and found that there were clear differences in viral replication and pathology between H2N2 isolates and infection routes. Importantly, the differences between H2N2 isolates in viral replication and pathology might be due to their varying binding preference for α2,3- and α2,6-SA.

## RESULTS

### HA sequence correlates with binding to α2,3- or α2,6-sialic acid.

**FIG 1:**
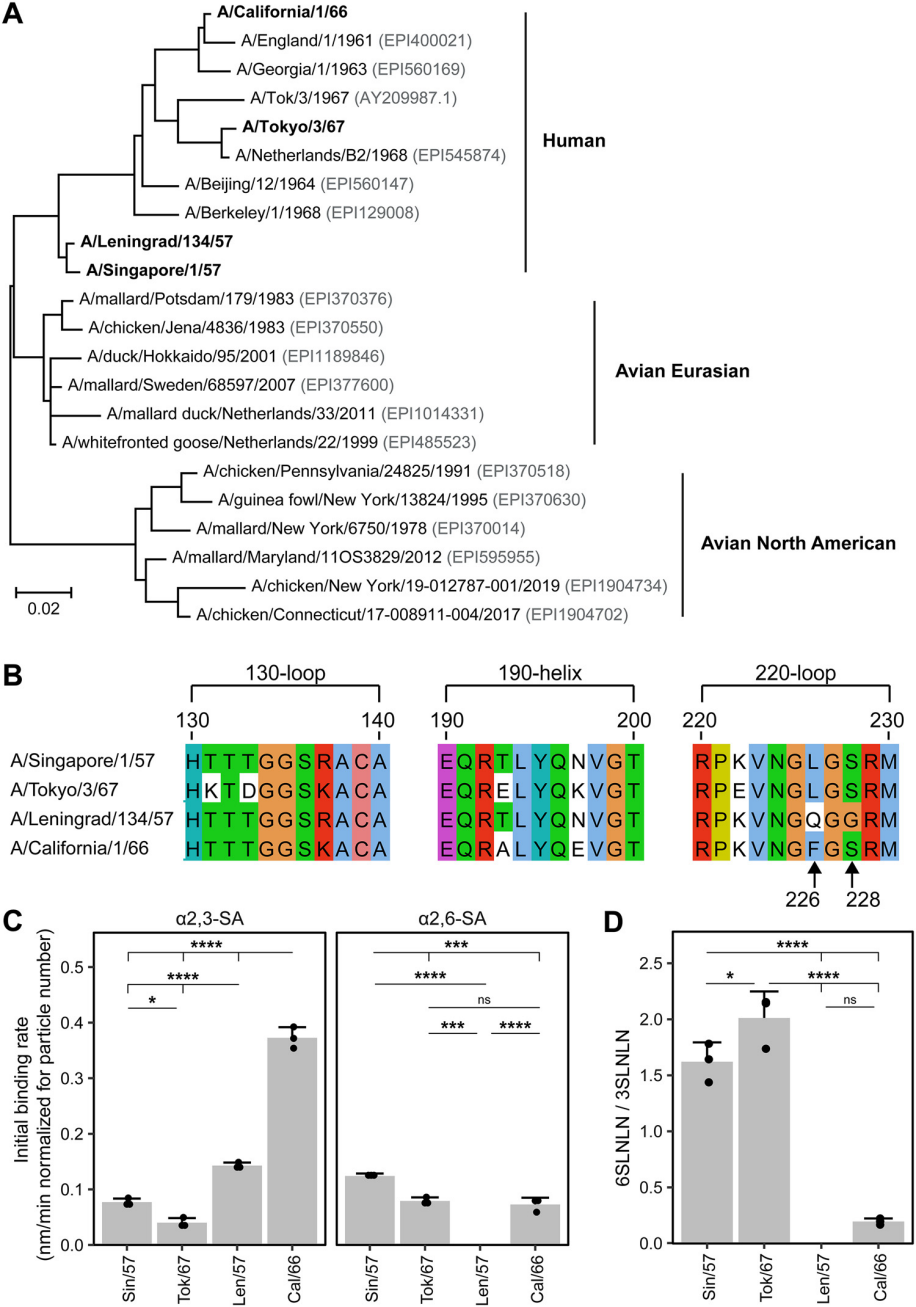
Binding of H2N2 strains to α2,3- and α2,6- sialic acid. (A) Phylogenetic tree of human and avian H2 sequences. The tree was constructed based on HA protein sequences by the maximum likelihood method. The four human H2N2 isolates used in this study are depicted in bold and the GISAID HA protein identifiers are written in gray. Scale bar depicts genetic distance. (B) Amino acid sequence alignment of the three sites involved in the binding to sialic acid (SA) (21) of H2 of the four human H2N2 influenza viruses used in this study. Colors indicate the (conservation of) amino acid profile based on the Clustal X color scheme. HA sequences are displayed according to H3-numbering (42). (C) Binding of H2N2 virus strains to α2,3- and α2,6-SA and (D) the binding ratio between α2,6- and α2,3-SA as determined by biolayer interferometry. The ratio of Leningrad could not be determined as binding to α2,6-SA was zero. Data is visualized as mean ± SD with *n* = 3. Binding rate of viruses in panels C and D was compared using 1-way ANOVA, followed by Tukey multiple-comparison test. *, *P* < 0.05; **, *P* < 0.01; ***, *P* < 0.001; ****, *P* < 0.0001. ns, not significant.

We selected two early and two late human H2N2 viruses for the infection of ferrets (Fig. 1A). A/Singapore/1/57 (Sin/57) and A/Leningrad/134/57 (Len/57) were both isolated during the pandemic in 1957. A/California/1/66 (Cal/66) and A/Tokyo/3/67 (Tok/67) are seasonal isolates from the end of H2N2 circulation and can be classified as clade I and II, respectively (4). These viruses display slight amino acid differences in their HA sequence that might affect their binding and replication properties. Due to this, these viruses may induce divergent pathological changes in the ferret model.

The binding preference of HA to sialic acid is primarily determined by the receptor binding pocket of HA, which is made up of the 130-loop, the 190-helix and the 220-loop (21). We sequenced the HA segments of the H2N2 isolates to investigate their binding preference. HA of Len/57 contains a Gln226 and Gly228 in the 220-loop (H3 numbering; Fig. 1B), which corresponds with the residues found in H2 derived from avian viruses. H2 HAs with these residues are predicted to bind to both avian-type α2,3-SA and human-type α2,6-SA (21). In contrast, Sin/57 and Tok/67 are probably better adapted to the human host as they contain Gln226Leu and Gly228Ser substitutions, which are known to result in strong preferential binding to α2,6-SA. While Cal/66 is similar to the latter two isolates in that it contains Ser228, it deviates with its Phe226. In addition, the H2 HAs of these viruses differ to some extent in their 130-loop and 190-helix, which is also likely to affect receptor binding specificity and/or affinity (Fig. 1B).

As binding preference markedly influences viral replication and consequently pathology and disease, we set out to confirm and further analyze the predicted binding properties of the selected viruses by biolayer interferometry. As this binding assay could only be performed under BSL-2 conditions, we used attenuated reassortant vaccine viruses of the respective BSL-3 classified original viruses. Sequence analyses of the vaccine viruses showed that while some mutations have arisen compared to wild-type viruses, none of these mutations are present in the HA-binding pocket (Table 1). The four viruses could all bind to α2,3-SA (Fig. 1C), although they differed to some extent in their initial binding rate. Most viruses—the exception being Len/57 with its avian-like HA (Gln226/Gly228)—also bound to α2,6-SA (Fig. 1C). Sin/57 and Tok/67 preferentially bound to α2,6-SA, in agreement with their human-like HA signature (Leu226/Ser228; Fig. 1D). While Cal/66 (Phe226/Ser228) was able to bind α2,6-SA, it preferred binding to α2,3-SA, just as Len/57, which did not display any binding to α2,6-SA. We conclude that the SA binding preferences of the different viruses largely correspond with their predicted preference based on the identity of the residues on position 226 and 228. Interestingly, while the Cal/66 virus was isolated many years after the start of the pandemic, it nevertheless prefers binding to avian-type receptors. It is important to note that most—if not all—H2N2 influenza virus isolates were initially propagated on eggs when they were first isolated, which might have introduced certain mutations in the HA binding domain to more efficiently bind α2,3-SA (22). This might explain why the relatively late pandemic Cal/66 virus displayed a preference for α2,3-SA, although this is difficult to verify.

**TABLE 1:** Mutations in HA of vaccine strain versus respective wild-type virus

Wild-type virus^*a*^	Vaccine virus^*b*^	Reference strain (HA accession #)^*c*^	Difference wild-type virus with vaccine virus (H3-numbering)	Difference wild-type virus with reference strain (H3-numbering)
A/Singapore/1/57 (GISAID: EPI2028789)	NIBRG-147 (GISAID: EPI2028785)	A/Singapore/1/57 (GISAID: EPI160146)	G158E	
A/Tokyo/3/67 (GISAID: EPI2028781)	A/17/Tokyo/67/326 (GISAID: EPI2028787)	A/Tokyo/3/67 (Genbank: AY209987.1)^*d*^	G126E	R81S; K93R; Y94D; S95G; G126R; K131T; D133T; K137R; Q145P; K259Q; I268M; C281F; N289K
		A/Netherlands/B2/1968 (GISAID: EPI545874)		P159Q; K186N; E193A; I436T
A/Leningrad/134/57 (GISAID: EPI2028783)	A/Leningrad/134/17/57 (GISAID: EPI2028784)	A/Leningrad/134/57 (GISAID: EPI555074)	V182M; N186T; V202I; I347V	I347V
A/California/1/66 (GISAID: EPI2028779)	A/17/California/66/395 (GISAID: EPI2028786)	A/California/1/66 (Genbank: AAO46291.1)^*d*^		A19S; M268L

a BSL-3 viruses used for ferret infections in this study.

b BSL-2 viruses used for biolayer interferometry in this study.

c Reference strain (same isolate or most closely related) derived from online depository to compare HA sequences with in-house HA sequence results of wild-type virus.

d Only partial reference sequence of HA is available.

We additionally investigated if the HA and NA protein sequences of the H2N2 isolates used in this study are identical to known reference sequences, since repeated passaging might have introduced mutations. No differences were detected in NA protein sequence and few amino acid mutations (Len/57 = 1; Cal/66 = 2) were present in the HA sequence of Sin/57, Len/57, and Cal/66 (Table 1). These mutations were not present in the receptor binding pocket of HA, and it is therefore unlikely that this influenced binding to sialic acid much. In the case of Tok/67, we found multiple differences between our virus isolate and the reference sequence (Table 1). However, according to a protein blast, the reference sequence is not similar to other (late) H2N2 clade II sequences and does not group together. In contrast, the Tok/67 HA protein sequence reported here is 99.12% similar to A/Netherlands/B2/1968 and clusters together with other H2N2 viruses (Table 1 and Fig. 1A). It is therefore plausible that the reference sequence is incorrect, while the Tok/67 sequence we report here is representative of late circulating clade II viruses given the high similarity to A/Netherlands/B2/1968.

### Viral replication and tissue distribution differs between H2N2 isolates and route of infection.

**FIG 2:**
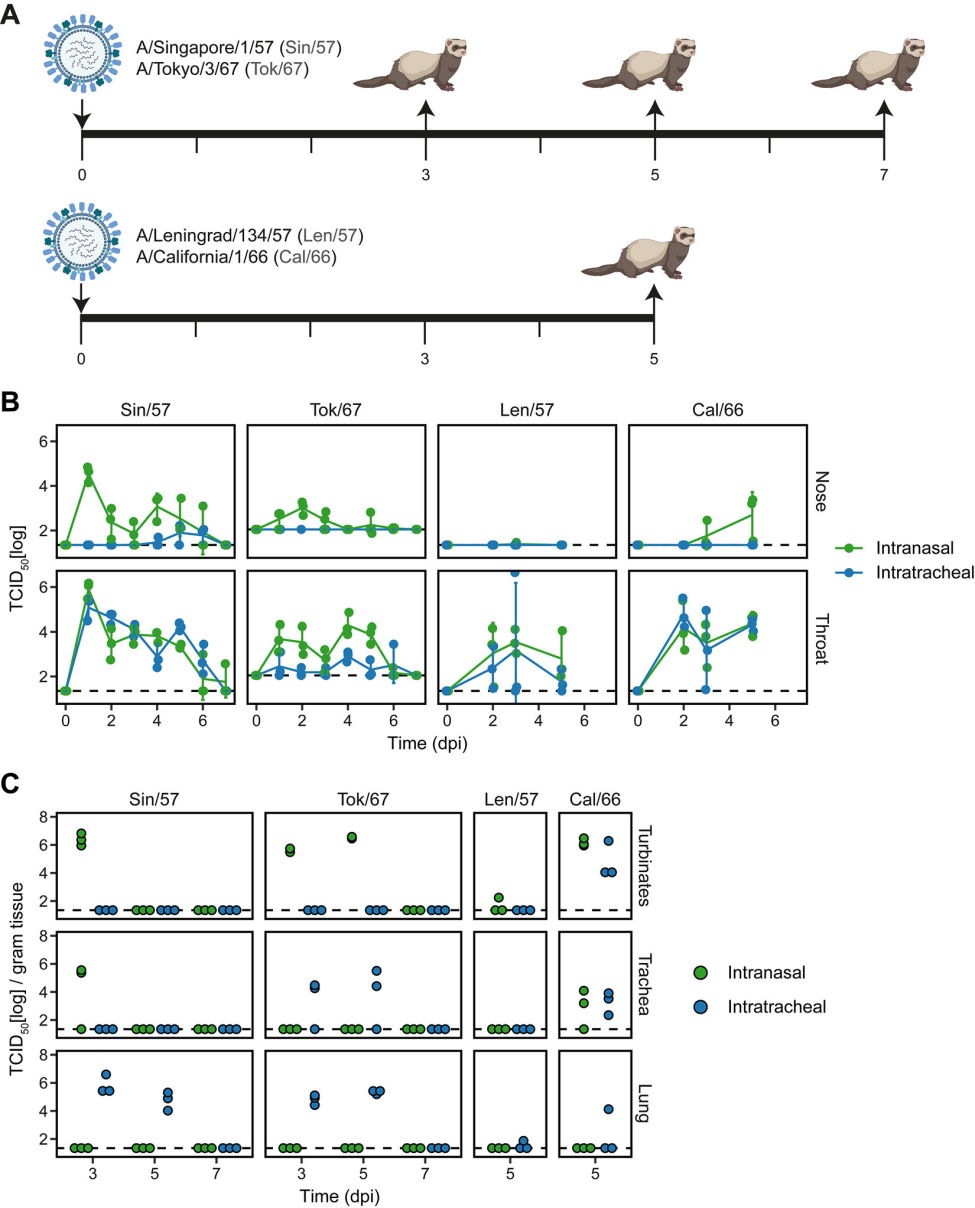
Virus tissue distribution and replication kinetics depend on the route of infection and virus isolate. (A) Female ferrets of 4–8 months old were infected with 10^6^ TCID_50_ influenza virus via intranasal or intratracheal inoculation on day 0. Animals were then euthanized on day 3, 5, 7 (Sin/57 and Tok/67) or only on day 5 (Len/57 and Cal/66) after infection to study viral replication and pathology. *n* = 3 per inoculation route and dissection day. Infections with Sin/57 and Tok/67 were carried out in separate experiments while Len/57 and Cal/66 infections were performed within one experiment. (B, C) Viral load in (B) nasal and throat swabs and (C) respiratory tissues was measured by TCID_50_-assay on MDCK cells. Dotted lines represent the limit of detection. Data are visualized as (B) mean ± SD or (C) individual values where each dot represents a ferret. dpi = days postinfection. “A” was created using BioRender.

Next, we performed a series of independent animal experiments in which we infected ferrets with the four H2N2 viruses and assessed the influence of i.n. versus i.t. inoculation on viral replication and pathology. We first infected ferrets with H2N2 isolates Sin/57 or Tok/67 and euthanized animals on 3, 5, and 7 days postinfection (dpi) to determine the kinetics of virus replication and development of pathology (Fig. 2A). Based on these experiments, we found that viral replication and pathology could be best assessed 5 dpi. Subsequent experiments with Len/57 and Cal/66 thus only assessed pathology and viral replication on 5 dpi.

We measured viral replication in the respiratory tract by TCID_50_ determination in nose and throat swabs. Similar to earlier reports for other H2N2 influenza viruses (15, 17), Sin/57 and Tok/67 replicated for approximately 6 days as most animals tested negative (below detection limit) on 7 dpi (Fig. 2B). Animals infected i.n. with Sin/57 clearly displayed higher viral titers in the nose than ferrets inoculated i.t., but no such difference was seen in the throat. In contrast, viral titers of i.t. infected animals with Tok/67 were lower in throat swabs and below detection limit in nasal swabs. Inoculation route did not influence the viral titers of Len/57 and Cal/66 in throat swabs, but viral titers in the nose did increase from 3 dpi onwards in ferrets infected i.n. with Cal/66. Importantly, no infectious virus could be detected in the nasal swabs of animals infected i.n. or i.t. with Len/57. In conclusion, with the exception of Len/57, viral replication in the nose was higher in i.n. infected animals for the isolates we investigated. The inefficient replication of Len/57 in the nose may be explained by the inability to bind to α2,6-SA (Fig. 1C). Viral replication in the throat was comparable between i.n. and i.t. infected animals with the exception of the Tok/67 virus.

To investigate the URT and LRT in more detail, we homogenized nasal, trachea and lung tissue on days 3, 5, and 7 after infection and determined the viral load by TCID_50-_assays. In agreement with the nasal swabs, i.t. infected animals mostly tested negative for influenza in the nasal turbinates (Fig. 2C). This was however not the case for Cal/66, as virus was also found in the nasal turbinates and trachea of both i.n. and i.t. infected animals. For other viruses, viral replication in the trachea was limited to i.n. (Sin/57) or i.t. (Tok/67) inoculated ferrets. Len/57 hardly replicated in any of the tissues, with a viral load just above detection level in a few animals. As expected, viral replication in the lung was only observed in i.t. infected animals, showing that i.n. inoculation is insufficient to establish an LRT infection with the H2N2 viruses tested. While Sin/57 and Tok/67 efficiently replicated in the lungs after i.t. inoculation, this was not the case for Len/57 and Cal/66.

### Fever and weight loss differ between virus isolates and inoculation routes.

**FIG 3:**
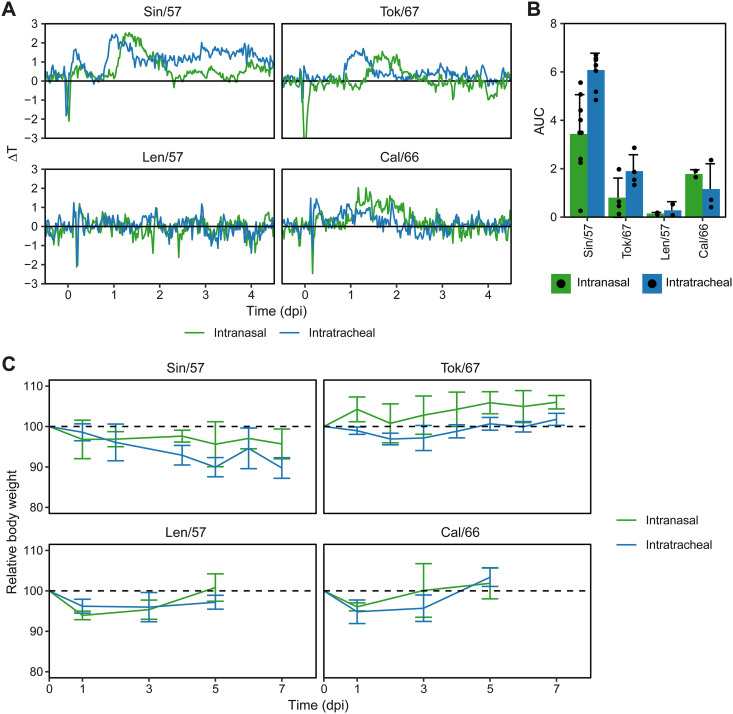
Fever and weight loss is dependent on virus isolate and route of infection. (A) Body temperature was measured in 30-minute intervals via an abdominal transponder. Data are visualized as deviation from baseline (ΔT), with lines indicating the mean per group. (B) Area under the curve (AUC) of data from “A” up to 5 days postinfection (dpi). Temperatures more than 2× SD below baseline were excluded as these are often due to anesthesia. Data are visualized as mean ± SD and individual values (black dots). *n* = 8–9 for Sin/57; 4 for Tok/67; 2 for Len/57; and 2–3 for Cal/66. (C) Body weight during infection relative to body weight at the day of infection. Data are presented as mean ± SD, with *n* = 3–6 for Sin/57 and Tok/67; *n* = 3 for Len/57 and Cal/66.

The clinical symptoms we observed were generally mild, with only a few Sin/57-infected animals displaying a minor reduction in activity and increased difficulty breathing. No pronounced clinical symptoms were observed for the infections with other virus isolates. We additionally measured fever as this is an unbiased measure for disease severity. In general, LRT infections are more severe (18–20), and this might thus affect the duration or height of fever. Tok/67 and Sin/57 both replicated in the LRT, and i.t. infection led to earlier onset of fever (Fig. 3A). Especially in the case of Sin/57, fever was longer-lasting for i.t. infected animals. Cal/66 infection induced a minor fever, but there was no strong difference between i.n. and i.t. infected animals. This is in agreement with the observed restriction of virus replication to the URT and failure to establish an LRT infection (Fig. 2B). Animals infected with Len/57 did not show any fever, independent of route of infection, which is also in line with the absence of an established infection.

The area under the curve (AUC)—which is a derivative of the sum of fever episodes within a certain timespan—confirmed that i.t. inoculation induced more severe fever for the preferentially α2,6-binding viruses Sin/57 and Tok/67 (Fig. 3B). This was not the case for Cal/66 or Len/57. These findings are corroborated by bodyweight data. For both Len/57 and Cal/66 viruses, weight decrease is similar between i.n. and i.t. infection (Fig. 3C). In contrast, Tok/67 and Sin/57 infections lead to a more severe decline in weight when they are administered i.t., although the within-group variation is relatively high. Weight loss was much more pronounced upon Sin/57 infection compared to Tok/67 infection.

### Pathology in the respiratory tract is influenced by the route of inoculation and virus isolate.

**FIG 4:**
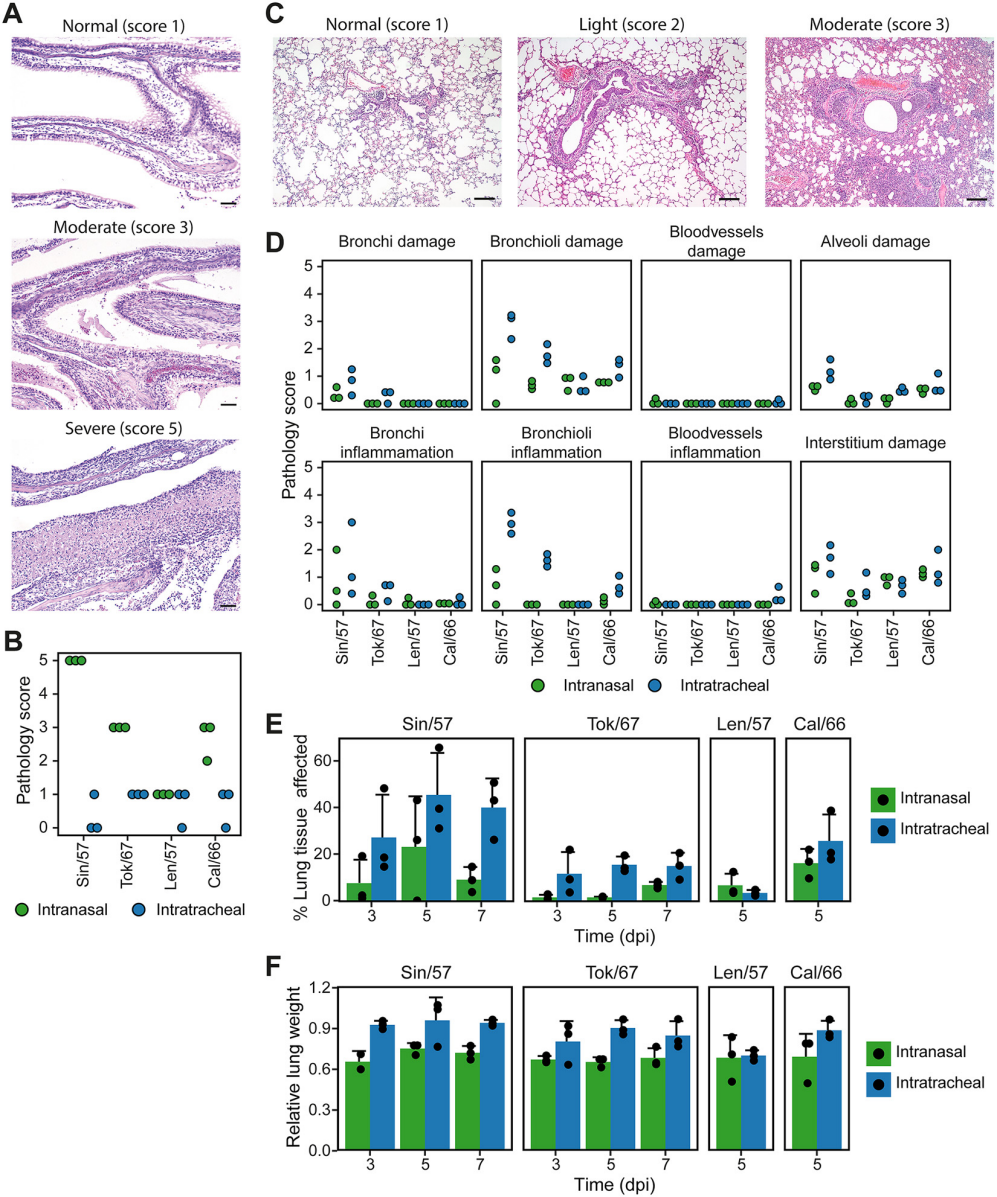
Pathology in respiratory tract is determined by virus isolate and route of inoculation. (A) Hematoxylin and eosin staining of representative nasal turbinate slides with different pathological severities at ×100 magnification. Bars represent 50 μm. (B) Pathological summary score (scale 0–5) of nasal turbinates 5 days postinfection (dpi). (C) Hematoxylin and eosin staining of representative lung slides with different pathological severities at ×50 magnification. Bars represent 200 μm. (D) Pathological scoring (scale 0–5) for damage and inflammation parameters of different segments of the lung at 5 dpi. (E) Percentage of lung tissue affected by pathology scored in D. (F) Lung weight after infection relative to body weight at the day of infection. Data are presented as individual values (A, C–E) with mean ± SD (D and E only). *n* = 3 for all plots, with the exception of Sin/57 in panel E (*n* = 2–3).

We observed that the virus isolate and route of infection affected the site of viral replication and clinical disease. In order to determine whether this also led to differences in pathological aberrations, we analyzed hematoxylin and eosin-stained slides of nasal turbinates and lung tissue at 5 dpi. The different pathology parameters scored in the nasal turbinates were summarized in a final pathology severity score on a scale of 0–5. As expected, the nasal turbinates were more severely affected in i.n. inoculated ferrets (score 1–5), as pathology was absent or mild in i.t. infected animals (score 0–1; Fig. 4A and B). I.n. infection resulted in aberrations of the naso- and maxilloturbinates, whereas the ethmoid (olfactory) turbinates were largely unaffected. At 5 dpi, a severe rhinitis was present in Sin/57 i.n. inoculated ferrets accompanied by hypertrophy of the goblet cells, pseudo squamous epithelium, and a severe (sub)mucosal inflammation. Together, this resulted in a pathology score of 5. Tok/67 infection was milder with a mild to moderate rhinitis and a minor inflammation of the submucosa. The respiratory epithelium was moderately affected over a large surface with hypertrophy and loss of cilia, resulting in a maximum score of 3. Ferrets infected i.n. with Cal/66 scored 2–3 and displayed aberrations in the surface of the respiratory epithelium ranging from minimal disturbances of the mucosa to pseudo desquamation of the epithelial lining. Inflammation and hyperemia were present in the submucosa. In contrast to the three other viruses, Len/57 did not cause much pathology in ferrets. Only slight disturbances and inflammation in the (sub)mucosa were observed, leading to a maximum score of 1 in both i.n. and i.t. Len/57 infected ferrets.

In the lungs, the infection induced a slight to moderate multifocal broncho-interstitial pneumonia of which the severity depended on the route of infection and virus isolate. The main pathological observations made throughout the groups are characterized by a multifocal inflammation around the terminal and respiratory bronchioles (peribronchiolitis) and ranges from a score 0–3 on a scale of 0–5 (Fig. 4C). The inflammation included lymphocytes, macrophages, and polymorphonuclear cells. In some cases, bronchiolar lumina were filled, but not obstructed, with mononuclear and polymorphonuclear infiltrate and some necrotic cellular debris. Disturbances to the epithelial lining of the bronchioli were restricted to a slight to moderate hypertrophy and hyperplasia. Sporadically, necrosis of bronchiolar epithelium and inflammation of the interstitium and hyperemia of the alveolar septa were observed. Alveolitis, alveolar hemorrhages, and perivasculitis were sometimes present. Sporadic aberrations were seen in the bronchi, and the bronchus was unaffected.

Multiple lung segments were scored for pathological parameters related to damage and inflammation on a scale of 0–5. Differences between infection routes and viruses were most prominent in the damage and inflammation of the bronchioli (Fig. 4D). As expected, lung pathology was more severe after i.t. inoculation, which was especially the case for Sin/57 and Tok/67. Little to no aberrations were detected in the bronchi and bronchioli of Len/57 and Cal/66-infected animals, reflecting the absence of clinical disease and viral replication in the LRT by these viruses. For all virus isolates except Len/57, we observed a trend that i.t. inoculation resulted in a higher percentage of affected lung tissue (Fig. 4E). We additionally determined the relative lung-to-body weight after H2N2 infection as an unbiased analysis of tissue inflammation and edema formation. The increased relative lung weights confirm that i.t. infection leads to more severe LRT pathology compared to i.n. inoculation (Fig. 4F). As expected, this was not the case for Len/57, which did not replicate in the LRT.

## DISCUSSION

Here we report our efforts to expand the ferret model for H2N2 influenza infection and disease for vaccine evaluation purposes. Of the four viruses we investigated, infection with Sin/57 and Tok/67—both of which prefer binding to α2,6-SA—lead to consistent high viral replication and disease symptoms. In contrast, Cal/66 replication was restricted to the upper respiratory tract and induced only mild disease. Len/57 infection hardly induced disease, reflecting the absence of an established infection. The site of replication for Sin/57 and Tok/67 was clearly dictated by the inoculation route, as virus deposited in the LRT did not infect the URT and vice versa. This is in contrast to Cal/66, where both URT and LRT inoculation resulted in replication only in the URT. Thus, for two viruses the site of replication was dictated by inoculation route, while for one virus the binding preference likely restricted replication to the URT. The site of replication also affected disease severity as an LRT-infection via i.t. inoculation led to more severe clinical disease and pathology when compared to i.n. inoculation.

Of the four H2N2 viruses that we tested, all infections were sublethal in ferrets, similar to the other (seasonal) human H1N1 and H3N2 viruses (23, 24). In contrast, infections with avian-derived H5N1 and H7N9 isolates are—depending on the strain and route of inoculation—lethal in ferrets (18, 19). The absence of mortality in this H2N2 influenza ferret model reflects the human situation during the H2N2 pandemic, which was not excessively deadly when compared to the 1918 pandemic or zoonotic infections with H5N1 and H7N9 (25, 26). Early reports indicated that the clinical symptoms of pandemic H2N2 disease did not differ much from regular seasonal influenza (reviewed in reference 27). Above all, most H2N2 pandemic deaths occurred in the very young, very old, or those with comorbidities (28). The most accurate representation of H2N2 infection should thus manifest itself as a mild, sublethal disease. In that aspect, both i.n. and i.t. inoculation of ferrets with H2N2 virus accurately mimics a human H2N2 infection.

It is difficult to compare the pathology in the H2N2 influenza ferret model with human cases, as pathology reports only document lethal H2N2 cases (27, 29, 30). However, based on these reports we found that the pathological facets in ferrets were similar to human cases, albeit less extensive. In both humans and ferrets, H2N2 infection could result in multifocal pneumonia (29). Hyperemia was present in ferrets, but seemed more severe in humans (29). The epithelial lining of the bronchioli was damaged in both humans (30) and ferrets, although for ferrets signs of damage were limited to hypertrophy and hyperplasia. Severe hyperemia, alveolar hemorrhage, and capillary thrombosis are indicative of a lethal infection and were only observed in human cases (29). The (severity of) pathology in ferrets was clearly influenced by the route of inoculation. In general, i.n. inoculation induced mild disease representative of a standard seasonal infection, while i.t. administration induced a moderate pneumonia. This was primarily the case for Sin/57 and Tok/67, which both replicated efficiently in the URT and LRT. Similar, but less severe, pathology was seen for Cal/66. In contrast, Len/57 replication in the LRT was below detection and similar to i.n. administration; i.t. inoculation did not induce disease or pathology.

We and other groups have infected ferrets with H2 influenza virus before. In a previous study, we infected ferrets with Cal/66 or Tok/67 only via the intranasal route (31). For both viruses, ferrets displayed robust viral replication in the throat and nasal turbinates. Although in this study a Cal/66 infection seemed to induce a slightly more severe fever compared to Tok/67 infection, pathology scores of the nasal turbinates of infected ferrets were similar between virus isolates and comparable to what we report here. Chen et al. inoculated ferrets i.n. and found that all viruses investigated replicated in both nasal turbinates and lung, despite i.n. administration (16). It is unlikely that this is due to differences in inoculation volume, but a higher infectious dose might have played a role. While Chen et al. used a higher infectious dose compared to us (10^7^ versus 10^6^ TCID_50_), their inoculation volume was smaller (0.2 vs 0.5 mL). Similarly, Moore et al. have shown that intranasal inoculation with 10^6^ TCID_50_ H1N1 or H3N2 influenza virus in a volume of 0.5 mL is sufficient to introduce virus into the lungs (32). Alternatively, the virus isolates tested by Chen et al. might have different SA-binding preferences or the viruses are less restricted by the inoculation route. Pappas et al. showed that H2N2 viruses with a preference for α2,3-SA transmit less efficiently in ferrets (15). For one virus, a Gln226Leu substitution naturally occurred in infected ferrets. This mutation is associated with enhanced binding affinity for α2,6-SA (33) and substantially improved transmission between ferrets (15, 17). This is likely a direct effect of the distribution of α2,3- and α2,6-SA in the ferret respiratory tract, where α2,6-SA is more abundantly expressed throughout the respiratory tract (34, 35). These studies and our results from the binding analysis offer some explanation as to why the α2,3-SA-binding Len/57 hardly replicates in ferrets and does not cause observable disease and pathology. Similarly, the α2,6-SA-prefering Sin/57 and Tok/67 are able to readily infect the ferret respiratory tract, leading to high viral titers and subsequent pathology. Cal/66 prefers α2,3-SA but can also still bind to α2,6-SA. Nevertheless, replication of Cal/66 in the lungs was much reduced compared to the α2,6-SA preferring viruses. Possibly, Cal/66 has adapted to replication at lower temperatures in the URT, which limits its replication in the warmer LRT. More research is needed to elucidate the molecular basis of this difference in replication.

Viral replication, disease and histopathology differed with inoculation route and virus. Clearly, Len/57 is not suitable to model H2N2 disease in ferrets due to the absence of productive viral replication and disease. Cal/66 in contrast did replicate and caused mild disease, although it was unable to infect the LRT. For a future ferret model, our preference would thus be to use either Sin/57 or Tok/67. Both viruses could replicate in the URT and LRT and were restricted by the inoculation route, which can be utilized to tweak the severity of disease. LRT infections induced by intratracheal inoculation tended to be more severe than intranasally-induced URT infections. In our opinion, intratracheal inoculation is therefore preferred for vaccine-challenge models assessing severity of disease, while intranasal administration would be more suitable to assess reduction of transmission by vaccine induced immunity.

With the study presented here, we show that ferrets are a representative model for human H2N2 influenza. The induced severity of disease and pathology can be altered by the route of infection and strain selection, enabling us to model both mild and moderate H2N2 disease. Together with the development of tools and reagents to study cellular and humoral immunity in ferrets (23, 36), we now have a working model to study vaccine-induced immune responses in the context of protection against influenza infection. These developments can further facilitate the research of new, improved influenza vaccines.

## MATERIALS AND METHODS

### Ethical statement.

All animal experiments were approved by the Animal Welfare Body of Poonawalla Science Park – Animal Research Center (Bilthoven, The Netherlands) under permit number AVD3260020184765 of the Dutch Central Committee for Animal Experiments. All procedures were conducted according to EU legislation. Animals were examined for general health on a daily basis and after infection, ferrets were scored daily for activity and impaired breathing. The following scoring system was used for activity: 0 = active; 1 = active when stimulated; 2 = inactive; and 3 = lethargic; and for respiratory distress: 0 = normal breathing; 1 = fast breathing; and 2 = heavy and stomach breathing. If animals showed severe disease according to the defined end points (lethargic or heavy breathing and inactive or more than 20% weight loss) prior to scheduled termination, they would be euthanized by cardiac bleeding under anesthesia with ketamine (5 mg/kg; Alfasan, Woerden, The Netherlands) and medetomidine (0.1 mg/kg; Orion Pharma, Espoo, Finland).

### Viruses.

Wild-type egg-grown H2N2 influenza viruses (A/Singapore/1/57, A/Leningrad/134/57, A/California/1/66, and A/Tokyo/3/67) with an unknown passage history were obtained from the influenza strain repository of the Institute of Experimental Medicine (IEM, St Petersburg, Russia). Live attenuated H2N2 viruses (A/Leningrad/134/17/57, A/17/California/66/395, and A/17/Tokyo/67/326) were likewise supplied by the IEM. The A/Singapore/1/57 reassortant (NIBRG-147, NIBSC code 09/306) virus was obtained from the National Institute for Biological Standards and Control (NIBSC, Hertfortshire, United Kingdom). All experiments involving wild-type H2N2 virus were carried out under BSL-3 conditions. Influenza viruses were grown on MDCK cells in MEM medium (Gibco; Thermo Fisher Scientific, Waltham, MA) supplemented with 40 μg/mL gentamicin, 0.01M Tricin, and 2 μg/mL TPCK treated trypsin (all from Sigma-Aldrich, Saint Louis, MO). At >90% cytopathic effect (CPE), the suspension was collected and spun down (4000 × *g* for 10 min) to remove cell debris and stored at –80°C. HA sequences of attenuated reassortant viruses were sequenced at Baseclear (Leiden, the Netherlands), and sequences are deposited in GISAID (identifiers in Table 1).

### Virus sequencing and alignment.

HA and NA segments of H2N2 influenza viruses were amplified by PCR with the MBTuni-12 [5′-ACGCGTGATCAGRAAAAGCAGG] and MBTuni-13 [5′-ACGCGTGATCAGTAGAAACAAGG]) primers (37). Sequencing was performed with the MinION (Nanopore Technologies, Oxford, United Kingdom), and sequence data were analyzed using an inhouse pipeline. HA sequences of wild-type human and avian influenza virus isolates were extracted from GISAID and GenBank (identifiers in Fig. 1A) (38, 39). Wild-type and reassortant H2N2 virus sequences were aligned by the MUSCLE algorithm using MEGA11 software (40). Aligned wild-type human H2N2 viruses were color-coded according to the Clustal X color scheme in Jalview 2.11.1.4 (41). These HA sequences are displayed according to H3-numbering (42). HA protein sequences of avian and human wild-type H2N2 viruses were used to construct a maximum likelihood phylogenetic tree in MEGA11. For all viruses depicted in the phylogenetic tree, HA protein sequences were derived from GISAID with the exception of A/California/1/66 and A/Tokyo/3/67, for which no full-length HA sequence has been deposited. Instead, the full-length HA sequences reported in this article have been used.

### Animal handling.

Female ferrets (Mustela putorius furo) supplied by Schimmel BV (The Netherlands) aged 4–8 months were tested for prior influenza and Aleutian disease infections, and only negative animals were selected. Upon arrival at the animal facility, ferrets were allocated into groups based on weight and housed by group in open cages. From the moment of infection, all procedures were carried out in BSL-3 certified isolators. A “DST micro T” temperature transponder (Star-Oddi, Garðabær, Iceland) was implanted intra-abdominally 14 days prior to commencement of the experiment to measure body temperature. For this procedure, animals were anesthetized with ketamine (5 mg/kg) and medetomidine (0.1 mg/kg). Buprenodale (0.2 mL; AST Farma, Oudewater, The Netherlands) was administered as a postoperative analgesic. Anesthesia by medetomidine was antagonized with atipamezole (0.25 mg/kg; Orion Pharma). Blood collection and infections were carried out with the same anesthetics, but for the latter atipamezole treatment was delayed by 30 min to avoid excretion of the inoculum by sneezing and coughing. Weight determinations and swabbing on days without other treatments (e.g., infection/blood draws) occurred under anesthesia with ketamine alone. The ferrets received food and water *ad libitum*. At scheduled termination, ferrets were euthanized by cardiac bleeding under anesthesia with ketamine and medetomidine.

### Study design.

The data presented here originated from three independent ferret experiments (1 = Sin/57; 2 = Tok/67; 3 = Len/57 and Cal/66). In each experiment, ferrets were infected intranasally (0.5 mL) or intratracheally (3 mL) with 10^6^ TCID_50_ of one of the four selected H2N2 influenza viruses. Three, 5, and 7 days after infection with A/Tokyo/3/67 and A/Singapore/1/57, animals were sacrificed in order to study pathology and virology. For experiments with A/Leningrad/134/57 and A/California/1/66, animals were euthanized 5 days postinfection only. Groups consisted of three animals per condition (route of infection and day of termination).

On 2, 3, and 5 days after infection, nasal and throat swabs were collected and bodyweight was measured. For A/Tokyo/3/67 and A/Singapore/1/57 infections, additional swabs and weight measurements were taken on days 1, 4, 6, and 7 postinfection. Nose and throat swabs were collected in 2 mL transport medium containing 15% sucrose (Merck, Kenilworth, NJ), 2.5 μg/mL Amphotericin B, 100 U/mL penicillin, 100 μg/mL streptomycin, and 250 μg/mL gentamicin (all from Sigma) and stored at –80°C. At predetermined time points, animals were dissected as described before (20). In short, animals were sedated (ketamine and medetomidine) and exsanguinated, after which the trachea was clamped off and the inflated lungs were isolated, weighed, and examined for gross pathology. The middle section of the trachea (~1 cm), sections of the three right lung lobes and the accessory lobe along the proximodistal axis (~1 cm by 3 mm), and the right nasal turbinates were isolated and stored in Lysing Matrix A tubes (MP Biomedicals, Irvine, CA) at –80°C for later virological analysis. The left cranial and caudal lung lobes and the left nasal turbinates were fixed in 10% buffered formalin for histopathological analysis.

### Animal temperature, bodyweight, and lung weight.

Temperature data were retrieved from the implanted temperature loggers and consisted of measurements taken every 30 min. Baseline temperature was calculated as the average temperature in the 4 days before infection. The change in temperature was calculated as deviation from baseline (ΔT). The area under the curve (AUC) was calculated as the total ΔT up until 5 dpi. Values smaller than “baseline–2*standard deviation of baseline” were excluded as these often occur due to anesthesia. Relative bodyweight and relative lung weight are expressed as a percentage of bodyweight or ratio on the day of infection.

### Virus quantification.

Thawed lung, trachea, and nasal turbinate samples were homogenized in Lysing Matrix A tubes using FastPrep (MP Biomedicals) and clarified by centrifugation for 5 min at 4000 × *g*. Nasal and throat swabs were thawed and vortexed. All samples for virus quantification were serially diluted and tested in sextuplicate on MDCK cells in infection medium (MEM + 40μg/mL gentamicin, 0.01M Tricin, and 2 μg/mL TPCK treated trypsin). CPE was scored after 6 days of culturing, and TCID_50_ values were calculated using the Reed–Muench method.

### Pathology.

Pathology scoring was performed as described before (43, 44). In brief, the left cranial and caudal lung lobes were inflated with, and stored in, 10% formaldehyde. After fixation, the lung lobes were embedded in paraffin and sliced into 5 μm thick sections. Slides were stained with hematoxylin and eosin and microscopically examined at ×50 or ×100 magnification. For each lung lobe, at least 6 microscopic fields were scored. Pathological scoring distinguished between the categories “epithelial damage” and “inflammation.” Damage related parameters included hypertrophy, hyperplasia, flattened or pseudo squamous epithelia, necrosis and denudation of bronchi(oli) epithelium, hyperemia of septa, and alveolar emphysema and hemorrhages. Inflammation related parameters included (peri)bronchi(oli)tis, interstitial infiltrate, alveolitis, and (peri)vasculitis characterized by polymorphonuclear (PMN) cells, macrophages, and lymphocytic infiltrate. Pathological findings were scored on a scale of 0 (no aberrations) to 5 (severe damage) and were summarized in two “end scores” for the categories “epithelial damage” and “inflammation.” The percentage of affected lung tissue was estimated at ×20 magnification.

Nasal turbinates were fixated and stained similar to lung tissue and analyzed as reported before (31). Slides were examined microscopically and a summary score (on a scale of 0–5) based on the severity and percentage of tissue affected by different histopathological parameters was determined. Histopathological parameters consisted of damage to the epithelial linings, presence of inflammatory cells, and the presence of exudate and/or hemorrhages. All microscopic slides were randomized and scored blindly.

### Receptor binding.

The binding activities of H2N2 viruses were analyzed by biolayer interferometry using the Octet RED348 (Fortebio, Fremont, CA), similarly as described previously (45, 46). Briefly, streptavidin sensors were loaded to saturation with biotinylated synthetic glycans 2,3-sialyl-N-acetyllactosamine-N-acetyllactosamine (3’SLNLN, referred to as α2,3-SA) or 2,6-sialyl-N-acetyllactosamine-N-acetyllactosamine (6’SLNLN, referred to as α2,6-SA). Synthetic glycans were synthesized at the Department of Chemical Biology and Drug Discovery, Utrecht University, Utrecht, the Netherlands. Subsequently, the sensors were moved to virus-containing wells in the presence of NA inhibitor oseltamivir carboxylate (OC) for 15–30 min to achieve virus binding curves, which were used for the determination of the virus initial binding rates (referring to vobs = dB/dT in nm/min) as described previously (46). All the experiments were conducted in PBS with calcium and magnesium at 30°C with sensor shaking at 1,000 rpm. Initial binding rates were normalized for virus particle numbers, which were determined by nanoparticle tracking analysis using a NanoSight NS300 instrument (Malvern Panalytical, Malvern, UK) as described previously (45). The virus preparations were prediluted with Ultra Pure PBS (Merck) to reach a concentration suitable for analysis with NTA. All measurements were carried out at 19°C. The NanoSight NS300 recorded five 60-s sample videos per analysis, which were then used for the analysis with the Nanoparticle Tracking analysis 3.0 software, generating the quantitative information on particle numbers. Particle numbers were used to determine the initial binding rate per particle.

### Data analysis, statistical testing, and data availability.

Data analysis and visualization was performed using R software (v4.0.2) (47) with the packages tidyverse (48), ggpubr (49), and ggplot2 (50). Virus titers were log_10_-transformed for visualization. Sialic acid binding data were compared using 1-way ANOVA, followed by Tukey multiple-comparison test in GraphPad Prism software (v9.1.0). No statistical testing was performed for ferret experiments as group numbers (*n* = 3) were insufficient for reliable statistical testing. Some data were excluded from analysis, which included temperature transponders that malfunctioned and shut down before the defined termination point. Lung weight was not measured for one A/Singapore/1/57-infected ferret at 3 dpi. No other data were excluded from analysis. Virus HA sequences are available from GISAID, and identifiers are displayed in Table 1 and Fig. 1. Data supporting the main figures is available upon request to the corresponding author.
